# Zoonotic *Cryptosporidium* spp. in Wild Rodents and Shrews

**DOI:** 10.3390/microorganisms9112242

**Published:** 2021-10-28

**Authors:** Rauni Kivistö, Sofia Kämäräinen, Otso Huitu, Jukka Niemimaa, Heikki Henttonen

**Affiliations:** 1 Department of Food Hygiene and Environmental Health, Faculty of Veterinary Medicine, University of Helsinki, FI-00790 Helsinki, Finland; sofia.kamarainen@fimnet.fi; 2 Natural Resources Institute Finland (Luke), FI-33720 Tampere, Finland; otso.huitu@luke.fi; 3 Natural Resources Institute Finland (Luke), FI-00790 Helsinki, Finland; jukka.niemimaa@luke.fi (J.N.); ext.heikki.henttonen@luke.fi (H.H.)

**Keywords:** *Cryptosporidium*, 18S rRNA gene, zoonosis, rodent, mouse, vole, shrew

## Abstract

There has been a significant increase in the number of reported human cryptosporidiosis cases in recent years. The aim of this study is to estimate the prevalence of *Cryptosporidium* spp. in wild rodents and shrews, and investigate the species and genotype distribution to assess zoonotic risk. Partial 18S rRNA gene nested-PCR reveals that 36.8, 53.9 and 41.9% of mice, voles and shrews are infected with *Cryptosporidium* species. The highest prevalence occurred in the *Microtus agrestis* (field vole) and *Myodes glareolus* (bank vole). Interestingly, bank voles caught in fields were significantly more often *Cryptosporidium*-positive compared to those caught in forests. The proportion of infected animals increases from over-wintered (spring and summer) to juveniles (autumn) suggesting acquired immunity in older animals. Based on Sanger sequencing and phylogenetic analyses, *Apodemus flavicollis* (yellow-necked mouse) is commonly infected with zoonotic *C. ditrichi*. Voles carry multiple different *Cryptosporidium* sp. and genotypes, some of which are novel. *C. andersoni*, another zoonotic species, is identified in the *Craseomys rufocanus* (grey-sided vole). Shrews carry novel shrew genotypes. In conclusion, this study indicates that *Cryptosporidium* protozoan are present in mouse, vole and shrew populations around Finland and the highest zoonotic risk is associated with *C. ditrichi* in *Apodemus flavicollis* and *C. andersoni* in *Craseomys rufocanus*. *C. parvum*, the most common zoonotic species in human infections, was not detected.

## 1. Introduction

In Finland and other Fennoscandian countries (Norway and Sweden), there has been a significant increase in the number of reported human cryptosporidiosis cases in recent years [1]. According to the Finnish Institute for Health and Welfare, the number of reported human cryptosporidiosis cases has increased by more than 40-fold since 2000; from four cases reported in 2000 (on average 12 cases per year from 2000 to 2010) to 571 cases in 2020 [2]. The majority of human cryptosporidiosis cases are caused by *C. hominis*, mainly transmitted from human to human and *C. parvum*, which is a zoonotic species and a common cause of diarrhea in calves, also in Finland. A small proportion of the human cases are caused by other *Cryptosporidium* species, which usually remain unidentified.

The genus *Cryptosporidium* has a wide genetic diversity, distribution and host range. There are over 30 identified *Cryptosporidium* species and several *Cryptosporidium* sp. genotypes. There is some degree of host specificity among *Cryptosporidium* species, but several of them may infect many different animal species, including humans. *Cryptosporidium* spp., which also cause human infections, are *C. felis*, *C. muris*, *C. meleagridis*, *C. cuniculus*, *C. viatorum*, *C. andersoni*, *C. scrofarum*, *C. canis*, *C. suis*, *C. ubiquitum* and *C. fayeri*, among others (for reviews see [3,4]).

Rodents are ubiquitous and shrews are also widespread and adapt successfully to a variety of environments. They often live in close proximity to humans and domestic animals on farms and may act as vectors for several zoonotic pathogens, including *Cryptosporidium* [5]. Many species infecting humans have also been detected in rodents [6,7,8,9,10]. Rodents are natural hosts for *C. muris* but *C. parvum*, *C. ubiquitum*, *C. tyzzeri*, mouse genotype II, *C. hominis*, *C. meleagridis*, *C. andersoni* and *C. viatorum,* among others, have also been detected. Novel species described from rodents include *C. alticolis* and *C. microti* from common voles [11], and *C. apodemi* and *C. ditrichi* from *Apodemus* spp. [6]. Of these, *C. ditrichi* has been recently associated with human infections in Sweden [12].

The prevalence of *Cryptosporidium* spp. in wild rodents and shrews differs between studies. In China, the *Cryptosporidium* prevalence among wild rodents was 6.8% [8], whereas in El Hierro, Canary Islands, Spain it was 48.6% [13]. It was 11% in wild rodents from Swedish pig and chicken farms [5], 25.8% from rural communities in Philippines [14], 35.5% in an urban area in Brazil [15], 34.2% in South Korea [16], 24.3% in Slovakia [7], 50.7% in USA and 12.1% in Europe [17]. Older studies [8,13] used sugar flotation, staining and microscopy in the detection of *Cryptosporidium* oocysts. More recently, mainly more sensitive PCR-based methods have been used [7,14,15,16,17]. From Finland, there is only one previous report on *Cryptosporidium* spp. in wild rodents [18]. In total, 172 Finnish wild rodents were examined with microscopic methods and *Cryptosporidium* oocysts were found from only two rodents; one of 131 *Microtus agrestis*, one of 41 *Myodes glareolus* and none of 43 *Alexandromys* (former *Microtus*) *oeconomus* samples. However, no subtyping was performed in that study.

The aim of this study is to estimate the prevalence of *Cryptosporidium* spp. in Finnish wild small mammals using nested-PCR based on the partial 18S rRNA gene and to further investigate *Cryptosporidium* species occurring in the samples, and assess their potential zoonotic risk based on the literature. *Cryptosporidium* spp. are found to be prevalent among rodents and shrews. Zoonotic species other than *C. parvum* are identified with the highest zoonotic potential associated with *C. ditrichi* in *Apodemus flavicollis* and *C. andersoni* in *Craseomys rufocanus*. Methodological considerations are also discussed.

## 2. Materials and Methods

### 2.1. Samples

Altogether 450 small mammals, representing 14 different rodent and shrew species (Table 1), were caught from forests and fields from different locations throughout Finland (Supplementary Dataset S1). Yellow-necked mice, all except for one sample, were collected from office and storage buildings from southern Finland during 2010–2015; the water vole was caught in 2014 and all other species in 2017 (May–June and September–November) by the Natural Resources Institute Finland (Luonnonvarakeskus, Luke) during their national regulatory monitoring. Dissected colons including fecal matter were stored in Eppendorf tubes at −20 °C or delivered fresh to the laboratory for further analyses. Yellow-necked mice and the water vole were stored frozen until thawed and dissected on the day of DNA extraction.

As a *Cryptosporidium*-positive control sample, a stool sample from a calf naturally infected with *C. parvum* was used.

### 2.2. DNA Extraction and Molecular Typing

DNA extraction was carried out using the DNeasy PowerSoil Kit (Qiagen, Hilden, Germany) following the manufacturer’s instructions, with the exception that the manufacturer recommends to use a maximum of 250 mg of sample material for DNA extraction and all the fecal matter available (in some cases including the intestine) of the small mammal colons, used as the starting material, was less than the recommended maximum amount. The positive control DNA from the calf’s stool sample was extracted using the same extraction kit. The DNA samples were stored at −20 °C for further analyses. The concentration and quality of the DNA were analyzed using the NanoDrop^®^ ND-1000 Spectrophotometer (Thermo Fisher Scientific, Waltham, MA, USA).

To detect *Cryptosporidium* DNA from the total extracted DNA, nested-PCR of the partial 18S rRNA gene was used. The master mix contained 1.25 U DreamTaq Green DNA polymerase (Thermo Fisher Scientific, Waltham, MA, USA), 1× buffer for DreamTaq, 0.4 μg/μL BSA, 200 μM dNTPs, 0.4 μM each primer and 5 μL of DNA template (primary PCR) or 2 μL of PCR product from the primary PCR (secondary nested-PCR) per 50 μL reaction balanced with PCR-grade water. Primers used in the primary-PCR were: forward SHP1 5′ ACC TAT CAG CTT TAG ACG GTA GGG TAT 3′ and reverse SHP2 5′ TTC TCA TAA GGT GCT GAA GGA GTA AGG 3′ [15]. Primers used in the secondary nested-PCR amplification were: forward SHP3 5′ ACA GGG AGG TAG TGA CAA GAA ATA ACA 3′ [15] and reverse SSU-R3 5′ AAG GAG TAA GGA ACA ACC TCC A3′ [19].

The PCR cycles included an initial denaturation of 3 min at 94 °C, followed by 35 cycles of denaturation for 45 s at 94 °C, annealing for 45 s at 55 °C (primary PCR) or 64 °C (secondary PCR) and an extension of 1 min at 72 °C, with a final extension of 7 min at 72 °C. If the band of the 18S rRNA gene PCR-product was very weak in gel electrophoresis, the second amplification was re-done using 40 cycles to increase the amount of amplified *Cryptosporidium* DNA. The PCR reactions were run either on an Axygen^®^ MaxyGene Thermal Cycler II (Corning, New York, NY, USA) or a S1000TM Thermal Cycler (Bio-Rad, Hercules, CA, USA). The PCR products were analyzed by gel electrophoresis run in TAE buffer in an ethidium bromide stained 1.5% agarose gel for 1.5 h at 100 V, and visualized using an AlphaImager Digital Imaging System (Alpha Innotech Corp., San Leandro, CA, USA).

The PCR products were purified using the GeneJET PCR Purification Kit (Thermo Fisher Scientific, Waltham, MA, USA) from the secondary nested-PCR, and subjected to Sanger sequencing using the same primers used in the secondary PCR. Sequencing reactions were performed at StarSEQ (Mainz, Germany). In case the sequencing failed the PCR products were first purified from the electrophoresis gels using the QIAquick Gel Extraction kit (Qiagen, Hilden, Germany) and if necessary further cloned into *E. coli* using the NEB PCR Cloning kit (*n* = 62) (New England BioLabs, Ipswich, MA, USA). The plasmids containing the correct secondary-PCR insert were extracted using the GeneJET Plasmid Miniprep kit (Thermo Fisher Scientific, Waltham, MA, USA), and the inserts were sequenced in both directions using the secondary PCR primers. The resulting forward and reverse sequences were aligned and assembled using the BioNumerics version 5.1 software (Applied Maths, Kortijk, Belgium).

### 2.3. Phylogenetic Analysis

The partial 18S rRNA gene sequences were compared to the nucleotide collection database (nr) using Standard Nucleotide BLAST (Natural Center for Biotechnology Information, Bethesda, MD, USA) (available at https://blast.ncbi.nlm.nih.gov/Blast.cgi, accessed on 28 June 2021), and all relevant *Cryptosporidium* spp. and genotype sequences were downloaded from GenBank to be included as references in the phylogenetic analyses (Supplementary Dataset S1). All the sequences were trimmed to the same length (486 bp equal to bases 492 to 977 for *Cryptosporidium parvum* isolate NEMC1 18S ribosomal RNA gene sequence, accession number AF222998) including the variable region of the partial 18S rRNA gene. MAFFT version 7 [20] sequence alignment server [21] (available at https://mafft.cbrc.jp/alignment/server/, accessed on 30 June 2021) was used to align the sequences with the L-INS-i iterative refinement method [22] using two iterations. The evolutionary history was inferred by the Maximum Likelihood method and Tamura 3-parameter model [23] implemented in MEGA X [24]. The percentage of trees in which the associated taxa clustered together is shown next to the branches (i.e., the bootstrap value from 1000 replicates). The initial trees for the heuristic search were obtained automatically by applying the Neighbor-Join and BioNJ algorithms to a matrix of pairwise distances estimated using the Maximum Composite Likelihood (MCL) approach, and then selecting the topology with superior log likelihood value. A discrete Gamma distribution was used to model evolutionary rate differences among sites. The rate variation model allowed for some sites to be evolutionarily invariable. The trees are drawn to scale, with branch lengths measured in the number of substitutions per site. CorelDRAW Graphics Suite 2020 (Corel Corporation, Ottawa, ON, Canada) was used for the final text editing of the consensus phylogenetic trees.

### 2.4. Sequence Availability

The nucleotide sequences produced in this study have been deposited in GenBank SUB10559292 under accession numbers OK605319–OK605535 (Supplementary Dataset S1).

### 2.5. Statistical Analyses

The SPSS Statistics 24 software (IBM, Chicago, IL, USA) was used for statistical analyses. Cross-tabulations were used to study the occurrence of *Cryptosporidium*-positive samples between different rodent and shrew species caught in different seasons, habitats, host sex and age groups. Chi-square tests were used to analyze the statistical significance of the cross-tabulated results. The result was considered statistically significant at the 5% risk level for *p*-values ≤ 0.05. Fisher’s exact test was used when less than five observations occurred in one or more cells of the table.

### 2.6. Ethics Statement

Animal trapping was carried out according to stipulations of national animal welfare and environmental legislature. According to the Finnish Act on the Use of Animals for Experimental Purposes (62/2006) and a further decision by the Finnish Animal Experiment Board (16 May 2007), the animal capture technique we used, i.e., using traps that instantly kill the animal, is not considered an animal experiment and therefore requires no animal ethics license from the Finnish Animal Experiment Board. All animal trapping took place with permission (MH5854/662/2011) on land owned by the Finnish Forest and Park Service or by permission by local landowners. A permit (7/5713/2013) for capturing protected species (all shrews are protected in Finland) was granted by the Finnish Ministry of the Environment.

## 3. Results

### 3.1. Prevalence of Cryptosporidium among Different Host Species

The majority (87.8%) of small mammals in this study represented four different species, namely *Myodes glareolus* (bank vole, *n* = 184), *Sorex araneus* (common shrew, *n* = 80), *Apodemus flavicollis* (yellow-necked mouse, *n* = 66) and *Microtus agrestis* (field vole, *n* = 65) (Table 1). The overall prevalence of *Cryptosporidium* sp. in Finnish small mammals was 49.1% based on the 18S rRNA nested-PCR (Table 1). The highest prevalence occurred in *Microtus agrestis* (67.7%), *Myodes glareolus* (56.5%) and *Sorex araneus* (43.8%). *Apodemus flavicollis* (36.4%) and *Alexandromys oeconomus* (tundra/root vole, *n* = 22, 36.4%) were also commonly infected with *Cryptosporidium* species. *Arvicola amphibius* (water vole, *n* = 1), *Microtus mystacinus* (former *Mi. levis*) (East European vole, *n* = 1), *Neomys fodiens* (Eurasian water shrew, *n* = 1), *S. minutus* (pygmy shrew, *n* = 2) and *S. caecutiens* (Laxmann’s shrew, *n* = 1), on the other hand, were all negative for *Cryptosporidium* species.

### 3.2. Impact of Season, Habitat, Host Sex and Age on Cryptosporidium Prevalence

The prevalence of *Cryptosporidium* was significantly higher in *Mi. agrestis*, *My. glareolus* and *S. araneus* during autumn compared to spring and summer (Table 2). Relatedly, both *My. glareolus* and *Mi. agrestis* juveniles and young subadults were significantly more often positive for *Cryptosporidium* spp. compared to over-wintered adults in spring. For *A. flavicollis* the collection dates were not known for all of the samples, only the year of collection, and thus the impact of season was not statistically significant possibly due to the low numbers of cases with known exact dates, compared to the other three species. Furthermore, *My. glareolus* trapped in fields were significantly more often *Cryptosporidium*-positive compared to those caught in forests. Sex was not so clearly associated with the *Cryptosporidium* status; however, in *A. flavicollis* females were significantly more often infected (*p* < 0.05). For the rest of the host species, data were either too scarce or no statistically significant differences were found.

### 3.3. Cryptosporidium Species and Genotype Distributions

**Figure 1:**
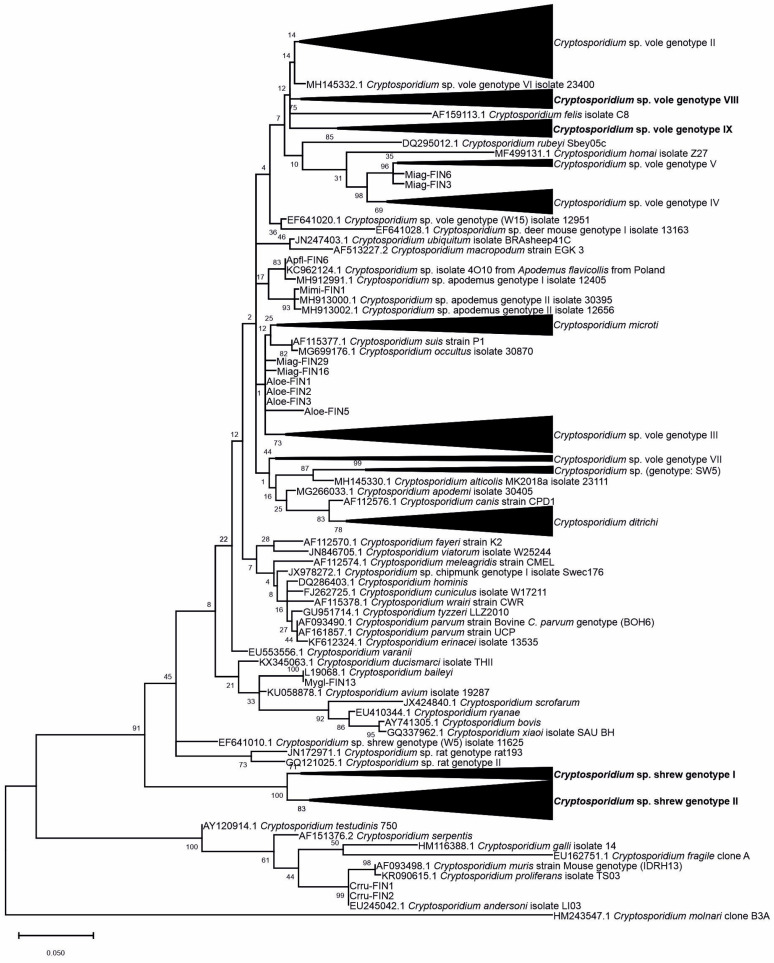
Phylogenetic Maximum Likelihood tree based on partial 18S rRNA gene sequences. The tree was rooted at midpoint. Bootstrap values of 1000 replications are shown at the branch nodes. Novel genotypes first described in this study are indicated in bold.

Partial 18S rRNA gene sequences were successfully obtained from most of the samples (Table 1). Only the *Myopus schisticolor* (wood lemming) nested-PCR product was revealed as a false positive based on the sequencing results. The Maximum Likelihood phylogenetic tree (Figure S1 and Figure 1) identified both previously described *Cryptosporidium* sp. and genotypes, and also the novel genotypes among the isolates (Supplementary Dataset S1). A summary of the results is presented in Table 1.

Voles carried the most diverse set of *Cryptosporidium* sp. and genotypes identified in this study (Table 1). Previously described species identified among our voles included *Cryptosporidium microti* from *Mi. agrestis* and *A. oeconomus*, *C. baileyi* from *My. glareolus* and *C. andersoni* from *C. rufocanus*. *C. microti* was identified from *Mi. agrestis* samples collected from seven different locations throughout southern and central Finland and *A. oeconomus* from one location in northern Finland (Supplementary Dataset S1). In addition, at least eight different genotypes were identified, out of which two were novel dominant genotypes from *Mi. agrestis* (vole genotypes VIII and IX). In *My. glareolus* the dominant genotypes were vole genotype II, III and IV. Part of the vole isolates did not form clear clusters and were thus only reported as *Cryptosporidium* sp. (Table 1 and Supplementary Dataset S1). In *C. rufocanus, C. andersoni* was the sole finding, as was *Cryptosporidium* sp. vole genotype III in *My. rutilus*.

*S. araneus* was infected with two novel shrew genotypes I and II that were clearly separated from previously described *Cryptosporidium* sp. and formed two separate clusters supported with high bootstrap values (Figure 1). Our only *S. minutus* isolate shared 99.58% sequence identity against isolate 05.1586.Va (GenBank sequence accession no. HM015878), previously described genotype SW4 from Scottish drinking water. The novel shrew genotype II was more prevalent in *S. araneus* (80.0%) (Table 1) and shared less than 97.1% sequence identity against genotype SW4. Both of the novel genotypes were found from shrews throughout Finland, often even simultaneously at the same sampling sites (Supplementary Dataset S1). Additionally, shrew genotype II was identified from one *My. glareolus* isolate from a same location from which a positive *S. araneus* was identified.

**Figure 2:**
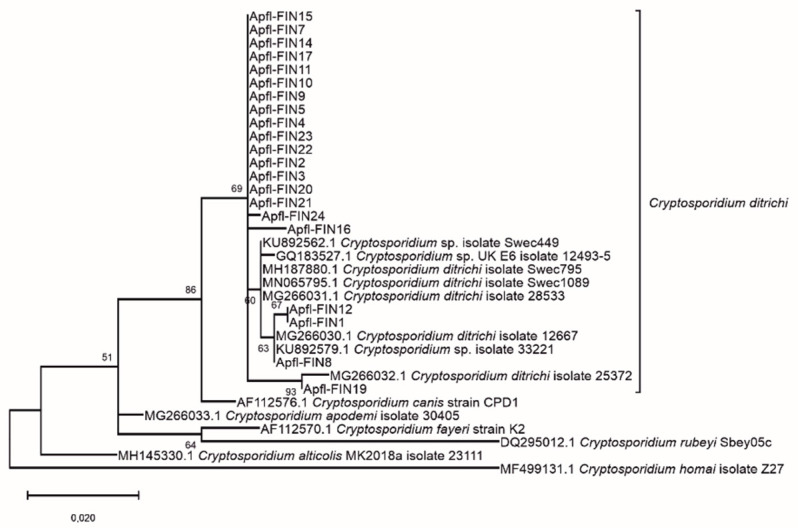
Phylogenetic Maximum Likelihood tree based on partial 18S rRNA gene sequences of isolates clustering with *Cryptosporidium ditrichi* (from Figure 1) and including additional reference sequences retrieved from GenBank. The tree was rooted at midpoint. Bootstrap values of 1000 replications are shown at the branch nodes.

The prevailing *Cryptosporidium* sp. identified in mice was *C. ditrichi*, accounting for 91.3% of the isolates from mice (Table 1), and was found from each of the sampled locations. Since the *C. ditrichi* isolates from *A. flavicollis* formed a quite diverse cluster in the phylogenetic analysis of the complete dataset (Figure 1 and Figure S1), a further phylogenetic analysis was performed, including all of the presumed *C. ditrichi* isolates and a larger and more diverse set of *C. ditrichi* reference sequences for comparison (Figure 2). All of the isolates clustered with *C. ditrichi* (sequence identity for isolate Apfl-FIN19 against the reference sequence GenBank accession number MG266030 was 98.57%), forming a cluster clearly separate from other previously described and closely related *Cryptosporidium* species with high bootstrap value. Our isolates were well among the known diversity of *C. ditrichi* and thus the preliminary species identification was confirmed. The minority of the *A. flavicollis* isolates and the sole isolate from *M. minutus* represented either previously described *Cryptosporidium* sp. apodemus I and vole II genotypes, or apodemus II genotype, respectively (Table 1).

## 4. Discussion

### 4.1. Prevalence of Cryptosporidium spp., and Effect of Season and Habitat

Nested-PCR of partial 18S rRNA gene from DNA samples extracted from intestinal samples from Finnish wild rodents and shrews revealed *Cryptosporidium* spp. prevalences of 36.8, 53.9 and 41.9% in mice, voles and shrews, respectively. Highest prevalences of 67.7 and 56.5% occurred in the *Microtus agrestis* (field vole) and *Myodes glareolus* (bank vole), respectively, followed by 43.8% in the *Sorex araneus* (common shrew) and 36.4% in the *Apodemus flavicollis* (yellow-necked mouse) and *Alexandromys oeconomus* (tundra/root vole). A systematic review and meta-analysis previously showed an overall global 17% prevalence of *Cryptosporidium* spp. infection in rodents [25]. Furthermore, previous studies have shown a prevalence of 13.7–31.8% in *Apodemus* spp. [7,26], 21.3–22.6% in voles [7,11] and 14.3% in shrews [7]. In a previous Finnish study using microscopic methods, wild voles were infected with *Cryptosporidium* spp. in 0.8% of *Microtus agrestis*, 2.4% of *Myodes glareolus* and none of the *Alexandromys oeconomus* [18]. Thus, in our study we found a significantly higher prevalence of *Cryptosporidium* spp. in various small mammal species. The higher prevalence observed is likely due to our optimized nested-PCR method originally developed by Silva et al. [15] that we used in PCR-detection instead of the nested-PCR originally developed by Xiao et al. [19], which has been widely used in other studies. The original paper describing the novel primers identified an approximately 2.5-fold difference between these two PCR methods when used for detecting *Cryptosporidium* spp. in rats and mice [15], with the new method being significantly more sensitive. Furthermore, to extract high quality DNA from rodents’ or insectivores’ fecal samples, we used a soil kit, which has been shown to be more efficient than stool kits (see e.g., [27]), because soil has similar PCR inhibitors as small mammal feces.

Interestingly, the bank voles caught in fields were significantly more often *Cryptosporidium*-positive compared to those caught in forests. Previously, a study on the distribution of *Cryptosporidium* in a drinking water resource revealed the highest oocyst flux in the area with the highest human and cattle density, and the lowest contamination in the forested sub-catchment region [28]. The prevalence of *Cryptosporidium* spp. further increased from spring and summer to autumn in most of the species included in our study. Previously, autumnal peaks have been reported in the prevalence of *C. parvum* in house mice, wood mice and bank voles [29], adult livestock, young livestock and small wild mammals [30] in the UK, and wild rural rodents in Poland [31,32]. In humans, both in Finland and other EU countries, the number of reported cryptosporidiosis cases is highest during autumn (August-November) [2,33]. Other studies have not found clear seasonal trends in, e.g., pigs [34]. A likely explanation is associated with higher rainfall in autumn and waterborne routes of spreading the infection in forests and fields. *Cryptosporidium* oocysts have been observed to survive in water for extended periods and several waterborne outbreaks of cryptosporidiosis have been described in humans (reviewed in [35]). Moreover, juveniles and subadults of *My. glareolus* and *Mi. agrestis* were significantly more often positive for *Cryptosporidium* spp., compared to over-wintered adults, suggesting acquired immunity may also play a part in infection dynamics. This could also partially explain the autumnal peak in prevalence, as the majority of individuals in autumn are juveniles and subadults, compared to over-wintered adults in spring. Furthermore, the population size and density increases from spring to autumn, increasing the number of possible contacts and further facilitating the spread of infections.

### 4.2. Zoonotic Species and Other Genotypes Occurring in Rodents and Shrews

Previous studies have reported *C. parvum*, and other zoonotic species, in many rodent species [26], especially in urban areas [7,9]. However, they have been quite an infrequent finding in rodents overall and it has been suggested that e.g., *C. parvum* infections, might be transient and short-term and occur following exposure to contaminated manure from ruminants [26]. Furthermore, it has been suggested that *C. alticolis* and *C. microti*, which are vole-species specific, might have been misidentified as *C. parvum* in studies merely based on microscopic evaluation [11]. In our study small mammals in Finland did not carry *C. parvum*. This may be partially due to the sparsely populated nature of the country and low numbers of livestock, especially cattle, per square km, as well as the fact that samples presented only wild animals caught further away from livestock farms. Future studies on rodents caught on, or in close proximity of, cattle farms could be useful to see if *C. parvum* is truly more prevalent in rodents caught on farms compared to those caught from the wild and the extent of the transient nature of the infection.

*Apodemus flavicollis* (yellow-necked mouse) was commonly infected by the zoonotic *C. ditrichi* (21 out of 23 strains). A previous pan-European study also revealed that *C. ditrichi* and apodemus genotypes, I and II, were the most prevalent species and/or genotypes across Europe in *A. flavicollis* [26]. Other species identified included *C. apodemi*, *C. microti*, *C. muris*, *C. parvum* and *C. tyzzeri* [26]. We also identified *Cryptosporidium* sp. vole genotype II from *A. flavicollis*. This genotype was the second most common species or genotype identified in *My. glareolus* and it is possible that this finding was just a result of the passive passage of oocysts ingested from the environment, in a habitat shared by these mouse and vole species, as has also been suggested earlier for *C. microti* [26]. A recent study showed that *C. ditrichi* infection occurred in humans in Sweden, causing typical symptoms of cryptosporidiosis [12]. In one case, they reported that the infection was likely transmitted from mice to a man. Furthermore, *A. flavicollis* has previously been shown to also shed higher numbers of *C. ditrichi* in their feces, compared to other *Cryptosporidium* species [6]. *A. flavicollis* is very common in many parts of Europe, including southern and central Finland, and typically enters human houses and summer lodgings, especially during autumn and early winter months for warmth. This causes an increased risk of infection by *C. ditrichi* in humans in Finland where the climate is pronouncedly seasonal. It would be highly recommended to wear protective clothing and a facemask while cleaning or renovating houses or other lodgings potentially also infested by rodents. In parallel, the infections caused by Puumala orthohantavirus have their seasonal peak in later autumn and early winter when *My. glareolus* (bank voles) invade human dwellings throughout Finland [36]. In our study *My. glareolus* mostly carried vole-specific *Cryptosporidium* spp. genotypes. However, out of 102 samples, one (1%) was found to be positive for *C. baileyi,* which is not generally considered as a zoonotic species, but has recently been identified in an immunocompetent patient in Poland associated with pulmonary hamartoma [37]. *C. baileyi* is mainly associated with birds and is recognized as an economically important pathogen that causes serious respiratory disease in chickens, against which there are no effective control measures currently available.

*C. andersoni* was the only species identified in 15.4% of *Craseomys rufocanus* (grey-sided vole) samples. *C. rufocanus* is common in northern Finland, north of the Arctic Circle, and occurs throughout the Scandinavian Mountain Range and northern parts in Fennoscandia, northern Russia and Siberia up until China, Mongolia, Korea and Japan. There are no previous reports on *Cryptosporidium* spp. in *C. rufocanus*. *C. andersoni* was originally isolated and described from domestic cattle (*Bos taurus*) and was shown not to be infective in mice [38]. It is the predominant *Cryptosporidium* species in bovines and can also affect their productivity. More recently, *C. andersoni* has also been identified from camel, wisent, hamster, takin, giant panda and American mink (reviewed in [39]), as well as horses [40]. Previously the zoonotic importance of *C. andersoni* has been considered minor [39]. However, recent studies from China and India have shown that *C. andersoni* was a predominant *Cryptosporidium* species, causing diarrhea in humans [41,42], suggesting that it may be an emerging (zoonotic) species at least in some regions. Since the density of cattle and livestock, except the semi-domesticated reindeer, in Northern Finland is low, and Lapland is frequently visited by hikers, drinking untreated surface waters contaminated by *C. andersoni* from wildlife may potentially pose a risk for zoonotic transmission in Finland, as well. *Cryptosporidium* spp. infections in reindeer in Finland should be investigated, as it is also possible that it is the major host of some *Cryptosporidium* spp. in northern Finland and *C. rufocanus* may just have ingested the oocysts instead of being infected. This is supported by a previous study which found many vole species to be resistant against *C. andersoni* infection [43].

*C. microti* was identified for the first time in *Microtus agrestis* (field vole) and *Alexandromys oeconomus* (tundra/root vole) in the present study. Previously it has been identified from the common vole (*Microtus arvalis*) [11]. However, there are no reports on zoonotic transmission or infections caused by *C. microti* in humans. Overall, voles carried multiple different *Cryptosporidium* spp. and genotypes, some of which were novel in our study and some previously identified in voles [11,17], or water (e.g., genotypes SW5, and UK E4 and E7) and a calf (genotype UK E7) [44,45]. This adds to the known diversity of *Cryptosporidium* in voles and highlights the fact that oocysts shed by voles may survive in drinking water and some even infect calves, however infrequently. As UK E7 and E4 were quite common among voles in our study, new vole genotypes VIII and IX were proposed to better reflect the host of these genotypes. On the contrary, we observed quite low diversity among *Cryptosporidium* spp. from the common shrew (*S. araneus*), with two novel shrew genotypes, I and II, identified among the samples. The only *S. minutus* isolate was nearly identical (99.58% identity) to genotype SW4, previously described from drinking water in the UK [44]. To our knowledge our study is the first to characterize *Cryptosporidium* genotypes from the common shrew in detail and based on our results the novel genotypes are likely to represent new species, yet to be described, since they form clearly separate clusters in the 18S rRNA gene tree.

### 4.3. Further Methodological Considerations

In our study, we found that cloning of the nested-PCR products was necessary before sequencing for a large proportion (27.6%) of the samples due to poor quality sequence of the secondary PCR product directly (55 samples) or low yield of product (six samples) from the nested-PCR. For a few of these samples we sequenced two different clones, and in some cases the different clones represented different species and/or genotypes of *Cryptosporidium*. Thus, simultaneous infections with multiple *Cryptosporidium* sp. and/or genotypes seem to be quite common in rodents and shrews, which has also been suggested by previous studies [8,46]. Only one sample from *Myopus schisticolor* (wood lemming) revealed to be a false positive, indicating high specificity of the nested-PCR used in this study for detecting a large variety of *Cryptosporidium* species in small mammal feces.

## 5. Conclusions

This study indicated that *Cryptosporidium* protozoan are present and common in mouse, vole and shrew populations around Finland. Furthermore, partial 18S rRNA gene sequences revealed that Finnish wild rodents and shrews are infected by several different *Cryptosporidium* species and genotypes, some of which have been shown to be zoonotic. Thus, wild rodents and shrews may act as a reservoir for zoonotic *Cryptosporidium* species infection transmission to humans and domestic animals, even though *C. parvum* or *C. hominis*, which are the most common causes of human infections in Finland, were not found.

## Figures and Tables

**Table 1:** *Cryptosporidium* sp. PCR-positive samples identified in different hosts, and their respective *Cryptosporidium* sp. or genotypes identified based on partial 18S rRNA gene sequences. Novel genotypes first described in this study are indicated in bold.

Host Species (Common Name)	Total No. (%) Samples	No. PCR-Positive Samples (%; CI 95%)	*Cryptosporidium* sp. or Genotype(s) Identified (No. Samples)
*Apodemus flavicollis* (yellow-necked mouse)	66 (14.7%)	24 (36.4%; 24.9–49.1%)	*Cryptosporidium ditrichi* (21); *Cryptosporidium* sp. apodemus genotype I (1), vole genotype II (1)
*Micromys minutus* (harvest mouse)	2 (0.4%)	1 (50.0%; 1.3–98.7%)	*Cryptosporidium* sp. apodemus genotype II (1)
*Alexandromys oeconomus* (tundra/root vole)	22 (4.9)	8 (36.4%; 17.2–59.3%)	*Cryptosporidium microti* (3); *Cryptosporidium* sp. vole genotype III (1); *Cryptosporidium* sp. (4)
*Arvicola amphibius* (water vole)	1 (0.2%)	0 (0%; 0–97.5%)	-
*Craseomys rufocanus* (grey-sided vole)	13 (2.9%)	2 (15.4%; 1.9–45.5%)	*Cryptosporidium andersoni* (2)
*Microtus agrestis* (field vole)	65 (14.4%)	44 (67.7%; 54.9–78.8%)	*Cryptosporidium microti* (11); *Cryptosporidium* sp. vole genotype II (1), vole genotype V (3), **vole genotype VIII (13), vole genotype IX (11)**; *Cryptosporidium* sp. (5)
*Microtus mystacinus* (East European vole)	1 (0.2%)	0 (0%; 0–97.5%)	-
*Myodes glareolus* (bank vole)	184 (40.9%)	104 (56.5%; 49.0–63.8%)	*Cryptosporidium baileyi* (1); *Cryptosporidium* sp. vole genotype II (51), vole genotype III (24), vole genotype IV (17), vole genotype VII (3), **vole genotype IX (1), shrew genotype II (1)**; *Cryptosporidium* sp. (genotype: SW5) (4)
*Myodes rutilus* (red vole)	9 (2.0%)	1 (11.1%; 0.3–48.3%)	*Cryptosporidium* sp. vole genotype III (1)
*Myopus schisticolor* (wood lemming)	1 (0.2%)	1 (100%; 2.5–100%)	-
*Neomys fodiens* (Eurasian water shrew)	1 (0.2%)	0 (0%; 0–97.5%)	-
*Sorex araneus* (common shrew)	80 (17.8%)	35 (43.8%; 32.7–55.3%)	***Cryptosporidium*** **sp. shrew genotype I (7), shrew genotype II (28)**
*Sorex caecutiens* (Laxmann’s shrew)	1 (0.2%)	0 (0%; 0–97.5%)	-
*Sorex minutus* (pygmy shrew)	4 (0.9%)	1 (25.0%; 0.6–80.6%)	*Cryptosporidium* sp. (genotype: SW4) (1)
Total	450	221 (49.1%; 44.4–53.8%)	

**Table 2:** Prevalence of *Cryptosporidium* sp. in *Mi. agrestis*, *My. glareolus* and *S. araneus* according to season, habitat, host sex and age.

	No. *Cryptosporidium* PCR-Positive/PCR-Negative Samples (%-pos.; CI 95%) per Host Species			
Variable	*Apodemus flavicollis*	*Microtus agrestis*	*Myodes glareolus*	*Sorex araneus*
Season (months)				
Spring/Summer (May–June)	6/15 (28.6%; 11.3–52.2%)	19/18 (51.4%; 34.4–68.1%)	9/67 (11.8%; 5.6–21.3%)	5/17 (22.7%; 7.8–45.4%)
Autumn (September–November)	10/7 (58.8%; 32.9–81.6%)	25/3 (89.3%; 71.8–97.7%)	96/12 (88.9%; 81.4–94.1%)	30/26 (53.6%; 39.7–67.0%)
Chi-square test *p*-value	0.0604	0.0012	<0.00001	0.0137
Habitat				
Field	NA	43/19 (69.4%; 56.4–80.4%)	47/6 (88.7%; 77.0–95.7%)	14/9 (60.9%; 38.5–80.3%)
Forest	NA	0/1 (0%; 0.0–97.5%)	52/32 (61.9%; 50.7–72.3%)	16/16 (50.0%; 31.9–68.1%)
Chi-square test *p*-value	ND	ND	0.0007	0.4246
Host sex				
Female	16/13 (55.2%; 35.7–73.6%)	23/13 (63.9%; 46.2–79.2%)	48/25 (65.8%; 53.7–76.5%)	7/10 (41.2%; 18.4–67.1%)
Male	8/29 (21.6%; 9.8–38.2%)	21/8 (72.4%; 52.8–87.3%)	56/54 (50.9%; 41.2–60.6%)	13/19 (40.6%; 23.7–59.4%)
Chi-square test *p*-value	0.0049	0.4650	0.0471	0.9702
Age				
Juvenile/Subadult	0/6 (0%; 0.0–45.9%)	21/2 (91.3%; 72.0–98.9%)	91/10 (90.1%; 82.5–95.2%)	1/0 (100%; 2.5–100.0%)
Adult/Over-wintered adult	23/36 (39.0%; 26.6–52.6%)	22/17 (56.4%; 39.6–72.2%)	13/69 (15.9%; 8.7–25.6%)	20/29 (40.8%; 27.0–55.8%)
Chi-square test *p*-value	ND	0.0040	<0.00001	ND

ND = not determined, NA = not applicable.

## Supplementary Materials

The following are available online at https://www.mdpi.com/article/10.3390/microorganisms9112242/s1, Dataset S1: Complete list of positive samples with their respective id, isolation source and partial 18S rRNA gene sequence accession numbers, and selection of reference sequences included for the phylogenetic analyses, Figure S1: Phylogenetic tree including all the isolates (Dataset S1) identified in this study.
