# Supplementary figures and images for: Zoonotic Cryptosporidium spp. in Wild Rodents and Shrews
Source: Microorganisms. 2021 Oct 28;9(11):2242. doi: 10.3390/microorganisms9112242 (PMC8618411; doi:10.3390/microorganisms9112242)

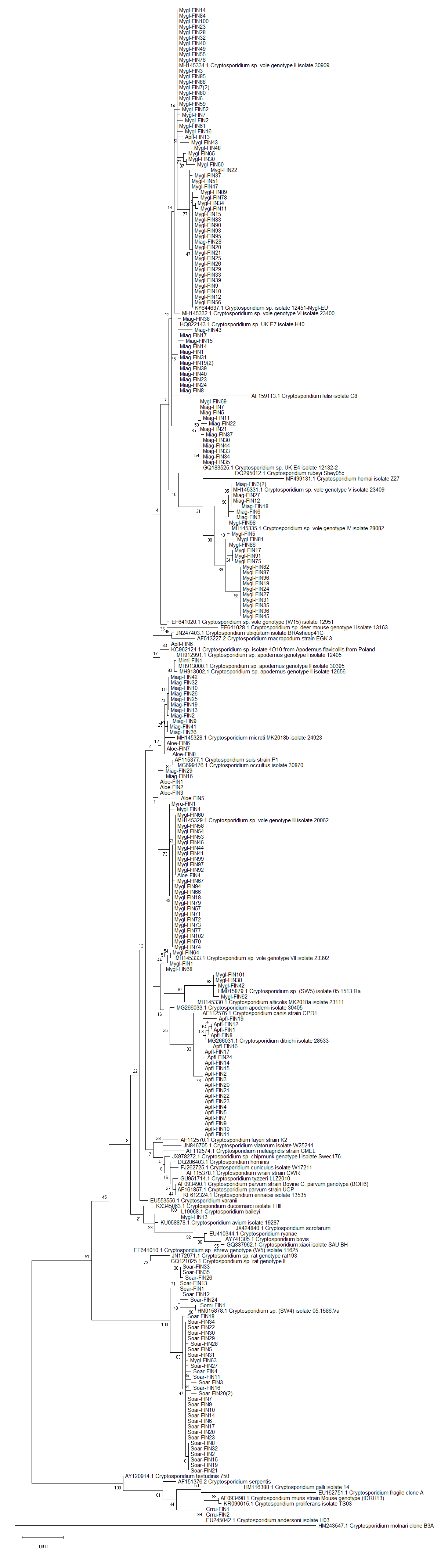

Supplement: Supplementary file 1 [file microorganisms-09-02242-s001.zip › Supplementary Figure S1.tif]
